# Associations of diabetes subgroups and lifestyle with incident dementia: insights from unsupervised phenotype clustering analysis

**DOI:** 10.3389/fendo.2026.1938222

**Published:** 2026-09-08

**Authors:** Xuan Zhao, Kan Wang, Zhiping Tan, Siyu Wang, Xi Meng, Yu Xiang, Yueyue Wang, Xiaoyun Zhang, Hong Lin, Shuangyuan Wang, Mian Li, Tiange Wang, Zhiyun Zhao, Jie Zheng, Min Xu, Jieli Lu, Weiqing Wang, Guang Ning, Yufang Bi, Yu Xu

**Affiliations:** 1 Department of Endocrine and Metabolic Diseases, Shanghai Institute of Endocrine and Metabolic Diseases, Ruijin Hospital, Shanghai Jiao Tong University School of Medicine, Shanghai, China; 2 National Clinical Research Center for Endocrine and Metabolic Diseases (Shanghai), Key Laboratory for Endocrine and Metabolic Diseases of the National Health Commission of the PR China, Shanghai Key Laboratory for Endocrine Tumor, State Key Laboratory of Medical Genomics, Ruijin Hospital, Shanghai Jiao Tong University School of Medicine, Shanghai, China

**Keywords:** clustering analysis, dementia, diabetes subgroups, lifestyle, self-organizing maps (SOMs)

## Abstract

**Background:**

To investigate heterogeneity in dementia risk across diabetes subgroups and examine whether lifestyle mitigates this risk.

**Methods:**

We did an unsupervised clustering analysis (self-organizing maps) in patients with diabetes from the Health and Retirement Study (HRS). Clustering variables included age at diabetes onset, body mass index (BMI), hemoglobin A1c (HbA1c), total cholesterol (TC), high-density lipoprotein cholesterol (HDL-c), systolic blood pressure (SBP), estimated glomerular filtration rate (eGFR), and C-reactive protein (CRP). Healthy lifestyle factors included never smoking, low to moderate alcohol consumption, and top tertile of physical activity. Incident dementia was defined as a self-reported diagnosis or Telephone Interview for Cognitive Status (TICS) scores ≤6.

**Results:**

We included 10,705 participants (40.2% men and mean age 67.6 years) in this analysis. Three subgroups were identified among diabetes: subgroup 1 (high BMI and elevated CRP), subgroup 2 (late-onset diabetes, high blood pressure, and low eGFR), and subgroup 3 (early-onset diabetes, high HbA1c, and high cholesterol levels). Compared to normoglycemia, subgroup 3 of diabetes showed a significantly higher risk of dementia (hazard ratio [HR] 1.43, 95% confidence interval [CI] 1.15-1.77), while the other subgroups did not. However, adopting a favorable lifestyle was associated with a reduced risk of dementia in participants with normoglycemia (HR 0.70, 95% CI 0.57-0.87), prediabetes (HR 0.67, 95% CI 0.50-0.90), and subgroup 1 of diabetes (HR 0.33, 95% CI 0.14-0.79).

**Conclusions:**

Diabetes-related dementia risk is heterogeneous, and benefits of lifestyle may vary by diabetes subgroups.

## 1 Introduction

Previous studies have elucidated the potential benefits of stratifying diabetes into different subgroups to develop tailored strategies for the prevention and treatment of certain diabetes complications, such as cardiovascular disease and chronic kidney disease (1, 2); however, whether this paradigm is also applicable to other complications (e.g., cognitive impairment) remains uncertain. Diabetes is associated with approximately a two-fold increased risk of dementia (3, 4). Currently, no pharmacological interventions have proven effective in curing dementia. Therefore, it is of paramount importance to direct attention towards preventive measures, with emphasis on identifying modifiable risk factors to delay or prevent the onset of dementia in diabetes.

Lifestyle factors are increasingly recognized as potential targets for dementia prevention (4, 5). In recent years, a growing body of observational studies has found that adherence to a combined healthy lifestyle is associated with a reduced risk of dementia (6, 7). Nevertheless, it remains unclear whether the risk of dementia varies across different diabetes subgroups and to what extent a healthy lifestyle can mitigate dementia risk in these subgroups. In this study, we used data from the Health and Retirement Study (HRS) to identify distinct diabetes subgroups, assess their related risk of dementia, and explore how combined healthy lifestyle factors influence dementia risk across these subgroups.

## 2 Methods

### 2.1 Study population

The HRS is a longitudinal panel study, conducted biennially since 1992, among community-dwelling individuals aged 51 years and over in the United States. Details regarding the study design and methods can be found elsewhere (8). Data collection comprised face-to-face (FTF) for most baseline interviews and telephone for primary follow-up interviews. A proxy respondent was sought for participants who were unwilling or unable to complete an interview. Until 2006, the HRS employed a mixed-mode design for follow-up, with half of the sample randomly assigned to FTF interviews for physical and biological measures, as well as psychosocial questionnaires, referred to as the enhanced FTF (EFTF) interview. This process alternated in subsequent waves, meaning the other half underwent EFTF interview in 2008. The HRS study was approved by the Institutional Review Board at the University of Michigan and the National Institute on Aging (HUM00061128). All participants provided written informed consent.

To maximize the sample size, individuals from the 2006 wave and 2008 wave who participated in EFTF were combined as the baseline in this study. Flowchart of study population is shown in **Supplementary Figure 1**. Overall, 9570 and 8296 participants were assigned to the EFTF interview in 2006 and 2008, respectively. Of these, 6735 and 6329 had available biomarkers data, respectively. Among these individuals, 6606 had normoglycemia, 3016 had prediabetes, and 3070 had diabetes. We further excluded individuals with diagnosed diabetes before age 18 (n = 15), and those with missing values for clustering variables (n = 763). Finally, 2292 participants with diabetes were included in the clustering analysis. For the analysis of incident dementia, we excluded 1209 participants with prevalent dementia or unavailable cognitive status at baseline, including 607 participants with normoglycemia, 305 with prediabetes, and 297 with diabetes, resulting in a final analysis sample of 10,705 participants.

### 2.2 Ascertainment of glycemic status

Since the HRS only collected data on hemoglobin A1c (HbA1c), we defined glycemic status based on HbA1c levels, along with disease history and medication use. Diabetes was defined as HbA1c of 6.5% or more, or a self-reported diagnosis of diabetes, or glucose-lowering medication use. In participants without diabetes, normoglycemia was defined as HbA1c of less than 5.7%, and prediabetes was defined as HbA1c between 5.7% and 6.4% (9).

### 2.3 Assessment of healthy lifestyle

Healthy lifestyle factors, including never smoking, low to moderate alcohol consumption, and top tertile of physical activity (PA), were chosen for the reason that they have been reported as modifiable risk factors for dementia prevention and can be altered at the individual level (4). We did not include diet because relevant dietary information was not collected at the baseline waves used in this study. Never smoking was defined based on the question “Have you ever smoked cigarettes” (yes or no). Alcohol consumption was assessed by the three-stage structured questions: “Do you ever drink any alcoholic beverages, such as beer, wine, or liquor?”; “In the last three months, on average, how many days per week have you had any alcohol to drink?”; and “In the last three months, on the days you drink, about how many drinks do you have?” Low to moderate alcohol consumption was defined as two drinks or fewer per day for men and one drink or fewer per day for women (10). PA score was calculated by multiplying the rounds of light, moderate, and vigorous PA per month by the metabolic equivalent (MET) weights for each intensity category and then summing them up (11). The frequency of “more than weekly” corresponded to 8.6 rounds/month, “weekly” corresponded to 4.3 rounds/month, “1–3 times per month” corresponded to 2.0 rounds/month, and “rarely/never” corresponded to 0 rounds per month (11). The MET weights of 2.3, 4.4, and 7.2 were assigned to light, moderate, and vigorous PA, respectively (11, 12), and the top tertile of the summed PA score was defined as the healthy level. We allocated 1 point for each healthy lifestyle factor and 0 points otherwise. The total lifestyle score, ranging from 0 to 3, was used to categorize participants as having an unfavorable (0 points), intermediate (1 point), or favorable (2–3 points) lifestyle. Since only 780 (7.3%) individuals scored 3 points, we combined those with scores of 2 and 3.

### 2.4 Assessment of incident dementia

In this study, participants were followed from baseline (2006 wave or 2008 wave) until 2020 wave, with cognitive assessments conducted biennially. We defined dementia using either a self-reported diagnosis or an alternative approach based on a cognition score. Cognitive function was assessed by the Telephone Interview for Cognitive Status (TICS), which includes immediate and delayed word recall tests, the serial-sevens test, and the counting-backward test (13). Composite TICS scores were calculated as the sum of individual cognitive test scores, ranging from 0 to 27, with higher scores indicating better cognitive function (13, 14). Incident dementia was defined as a self-reported diagnosis or TICS scores ≤6 during follow-up assessment (14, 15).

### 2.5 Statistical analysis

The analysis included two steps. In the first step, we aimed to identify subgroups with distinct characteristics among individuals with diabetes. To define multivariable subgroups of diabetes, we employed the self-organizing maps (SOM) method to identify subgroups with distinct characteristics among individuals with diabetes. SOM, an unsupervised technique based on Kohonen’s neural networks, is commonly used for dimensionality reduction and clustering analysis (16). The final output of SOM is visualized as simplified two-dimensional maps, which are divided into multiple regions. Individuals with similar profiles are located close to each other, while those with dissimilar profiles are positioned far apart. The coloring of each region reflects the average observed values of individuals within it, highlighting the clustering structure of the input data (17).

Clustering variables included age at diabetes onset, body mass index (BMI), HbA1c, total cholesterol (TC), high-density lipoprotein cholesterol (HDL-c), systolic blood pressure (SBP), estimated glomerular filtration rate (eGFR), and C-reactive protein (CRP), which are readily available at clinical settings and play crucial roles in the onset and progression of diabetes and its link to dementia (18–25). CRP was log-transformed due to its skewed distribution. We conducted the SOM analysis based on sex-adjusted data, in which each variable was standardized to eliminate differences between sexes. The analysis was conducted using the “*Numero*” package in R. Following the approach described by Bonnefous et al. (26), we employed model-based clustering analysis to validate the number of clusters derived from SOM, and the grouping with the highest Bayesian Information Criterion (BIC) was chosen as the final result. To further assess the robustness of the selected number of clusters, we performed an additional cluster validation using the NbClust consensus method (27).

In the second step, we aimed to evaluate the association of different glycemic status and diabetes subgroups with incident dementia, and to examine how lifestyle influenced dementia risk across these groups. Baseline characteristics of participants were described across normoglycemia, prediabetes, and the identified diabetes subgroups. Continuous variables are expressed as mean ± standard deviation (SD) or median (interquartile range [IQR]), and categorical variables are expressed as n (%). Differences among them were compared using one-way analysis of variance (ANOVA) test or Kruskal-Wallis test for continuous variables and chi-squared test for categorical variables, as appropriate.

The cumulative incidence of dementia was estimated using Kaplan-Meier curves across normoglycemia, prediabetes, and the identified diabetes subgroups, with the between-groups difference tested by the log-rank test. We used Cox proportional hazards models to estimate the hazard ratios (HRs) and 95% confidence intervals (95% CIs) for the related dementia risk across these groups with normoglycemia treated as the reference. The proportional hazards assumption was checked using Schoenfeld residuals. Person-years were calculated from baseline to the date of incident dementia, death, loss to follow-up, or the end of follow-up, whichever occurred first. Model 1 adjusted for age, sex, race, education level, marital status, hypertension, heart disease, stroke, lung disease, and cancer. Model 2 further adjusted for smoking status, alcohol consumption, and physical activity. The percentage of participants with missing covariates was only 1.3%; therefore, we used mean and median imputation to handle the missing values for these covariates. We further evaluated the association between categorized healthy lifestyle and dementia incidence across different diabetes subgroups, and checked for possible linear trend by treating healthy lifestyle score as a continuous variable.

We conducted several sensitivity analysis. First, we utilized the Fine-Gray competing risk regression model, taking death into account as a competing risk, to compute the subdistribution hazard ratio (SHR). Second, we excluded events of incident dementia that occurred within the first three years to reduce reverse causation. Third, we applied inverse probability of censoring weighting (IPCW) to account for potential informative censoring due to loss to follow-up. Fourth, we additionally adjusted for depression, apolipoprotein E (APOE)-4 carrier status and baseline TICS to assess the influence of residual confounding. Finally, we used multivariable-adjusted Cox regression with Firth’s penalized likelihood to alleviate bias arising from sparse events.

All statistical analyses were conducted using R software (version 4.3.1, The R Project for Statistical Computing, Vienna, Austria). A two-sided *P* value of less than 0.05 was considered statistically significant.

## 3 Results

### 3.1 Characteristics of participants in diabetes subgroups

**Figure 1:**
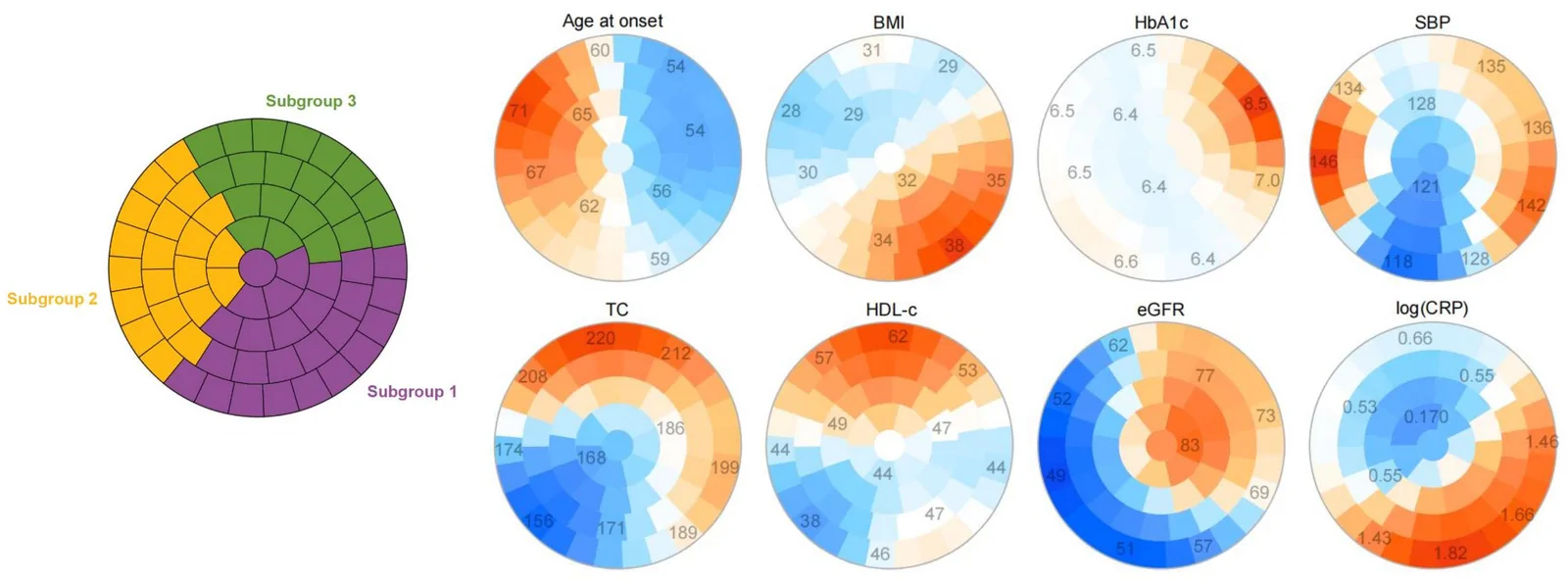
Results of SOM analysis. Based on the self-organizing maps (SOM), participants were divided into three subgroups (Left). Individuals with similar profiles are located close to each other, while those with dissimilar profiles are positioned far apart (Right). The coloring of each region was based on the average observed values of individuals within that region, revealing the clustering structure of input data. The red color indicates the highest average values, and the blue color indicates the lowest average values.BMI, body mass index; HbA1c, hemoglobin A1c; SBP, systolic blood pressure; TC, total cholesterol; HDL-c, high-density lipoprotein cholesterol; eGFR, estimated glomerular filtration rate; CRP, C-reactive protein.

**Figure 2:**
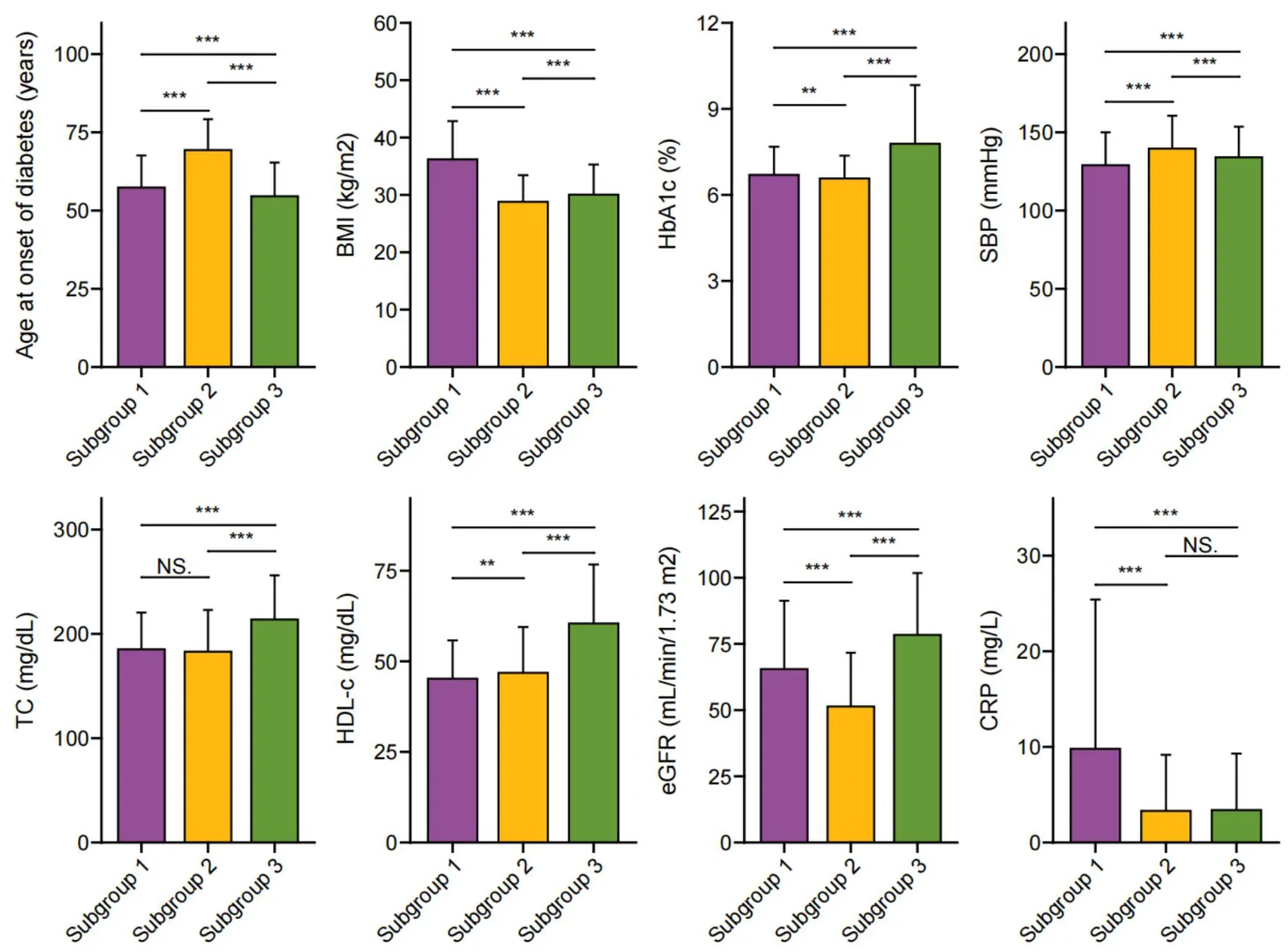
Characteristics of clustering variables in each diabetes subgroup. Subgroup 1: high BMI and elevated CRP. Subgroup 2: late-onset diabetes, high blood pressure, and low eGFR. Subgroup 3: early-onset diabetes, high HbA1c, and high cholesterol levels. BMI, body mass index; HbA1c, hemoglobin A1c; SBP, systolic blood pressure; TC, total cholesterol; HDL-c, high-density lipoprotein cholesterol; eGFR, estimated glomerular filtration rate; CRP, C-reactive protein. **P < 0.01; ***P < 0.001; NS, not significant. Data are expressed as mean and standard deviation.

In the clustering analysis of 2292 patients with diabetes, the SOM identified three subgroups (**Figure 1**). Detailed characteristics are provided in **Supplementary Table 1** and **Figure 2**. Subgroup 1, including 898 (39.2%) of the 2292 clustered patients, was characterized by high BMI and elevated CRP. Subgroup 2, including 731 (31.9%) patients, was characterized by late-onset diabetes, high blood pressure, and low eGFR. Subgroup 3, including 663 (28.9%) patients, was characterized by early-onset diabetes, high HbA1c, and high cholesterol levels. Model-based clustering and NbClust consensus method both supported a solution with three clusters was optimal (**Supplementary Figures 2**, **3**), consistent with the number of clusters determined by the SOM method.

### 3.2 Association of glycemic status and diabetes subgroups with incident dementia

A total of 10,705 participants were included in survival analysis after excluding those with prevalent dementia or unavailable cognitive status at baseline. The participants had a mean (SD) age of 67.6 (10.4) years, and 6400 (59.8%) were women. Among them, 5999 (56.0%) had normoglycemia, 2711 (25.3%) had prediabetes, 784 (7.3%) were in diabetes subgroup 1, 631 (5.9%) were in diabetes subgroup 2, and 580 (5.4%) were in diabetes subgroup 3 (**Table 1**).

**Table 1:** Baseline characteristics of study participants according to different glycemic status and diabetes subgroups.

					Diabetes	
Characteristics	Total population	Normoglycemia	Prediabetes	Subgroup 1^*^	Subgroup 2^†^	Subgroup 3^‡^
	(n = 10,705)	(n = 5999)	(n = 2711)	(n = 784)	(n = 631)	(n = 580)
Age (years)	67.6 (10.4)	66.6 (10.7)	69.1 (10.3)	65.7 (8.6)	76.2 (7.7)	65.3 (7.8)
Female	6400 (59.8)	3588 (59.8)	1701 (62.7)	426 (54.3)	362 (57.4)	323 (55.7)
White	8994 (84.0)	5280 (88.0)	2234 (82.4)	578 (73.7)	527 (83.5)	375 (64.7)
Education						
Less than high school	1868 (17.4)	861 (14.4)	506 (18.7)	191 (24.4)	151 (23.9)	159 (27.4)
High school or equivalent	6405 (59.8)	3609 (60.2)	1632 (60.2)	466 (59.4)	379 (60.1)	319 (55.0)
College or above	2432 (22.7)	1529 (25.5)	573 (21.1)	127 (16.2)	101 (16.0)	102 (17.6)
Married	6933 (64.8)	4021 (67.0)	1692 (62.4)	513 (65.4)	335 (53.1)	372 (64.1)
Never smoking	4560 (42.6)	2524 (42.1)	1193 (44.0)	306 (39.0)	277 (43.9)	260 (44.8)
Low to moderate alcohol consumption	4337 (40.5)	2614 (43.6)	1081 (39.9)	228 (29.1)	216 (34.2)	198 (34.1)
Top tertile of physical activity	3568 (33.3)	2297 (38.3)	840 (31.0)	148 (18.9)	112 (17.7)	171 (29.5)
BMI (kg/m^2^)	29.3 (6.1)	28.3 (5.7)	29.5 (6.0)	36.3 (6.7)	28.9 (4.6)	30.1 (5.2)
HbA1c (%)	5.8 (0.9)	5.3 (0.3)	6.0 (0.2)	6.7 (1.0)	6.6 (0.8)	7.7 (2.0)
SBP (mmHg)	130.8 (20.4)	129.6 (20.1)	131.2 (20.4)	129.3 (20.5)	139.6 (20.4)	134.0 (19.6)
TC (mg/dL)	202.6 (41.7)	206.2 (41.5)	202.3 (41.7)	185.1 (34.9)	181.5 (38.0)	214.0 (42.8)
HDL-c (mg/dL)	54.9 (16.2)	57.1 (16.8)	54.3 (15.2)	45.1 (10.5)	46.9 (12.7)	60.6 (16.4)
eGFR (mL/min/1.73 m^2^)	73.4 (24.6)	76.5 (23.9)	72.1 (24.0)	67.1 (25.5)	52.1 (20.0)	78.5 (23.1)
CRP (mg/L)	2.1 (1.0-4.7)	1.8 (0.9-4.2)	2.3 (1.1-4.9)	5.4 (2.3-10.8)	1.6 (0.8-3.7)	1.7 (0.8-3.7)
Hypertension	7120 (66.5)	3598 (60.0)	1877 (69.2)	656 (83.7)	542 (85.9)	447 (77.1)
Heart disease	2346 (21.9)	1121 (18.7)	590 (21.8)	258 (32.9)	248 (39.3)	129 (22.2)
Stroke	674 (6.3)	319 (5.3)	162 (6.0)	76 (9.7)	82 (13.0)	35 (6.0)
Lung disease	934 (8.7)	464 (7.7)	257 (9.5)	108 (13.8)	57 (9.0)	48 (8.3)
Cancer	1488 (13.9)	766 (12.8)	406 (15.0)	119 (15.2)	140 (22.2)	57 (9.8)
Healthy lifestyle score^§^						
0	2515 (23.5)	1302 (21.7)	604 (22.3)	274 (34.9)	186 (29.5)	149 (25.7)
1	4673 (43.7)	2471 (41.2)	1273 (47.0)	362 (46.2)	302 (47.9)	265 (45.7)
2	2759 (25.8)	1714 (28.6)	661 (24.4)	124 (15.8)	126 (20.0)	134 (23.1)
3	758 (7.1)	512 (8.5)	173 (6.4)	24 (3.1)	17 (2.7)	32 (5.5)

^*^ Subgroup 1: high BMI and elevated CRP.

^†^ Subgroup 2: late-onset diabetes, high blood pressure, and low eGFR.

^‡^ Subgroup 3: early-onset diabetes, high HbA1c, and high cholesterol levels.

^§^ Healthy lifestyle score is based on three healthy lifestyle factors: never smoking, low to moderate alcohol consumption, and top tertile physical activity, with 1 point per healthy lifestyle factor.

Data are mean (SD) or median (IQR), and n (%) for continuous variables and categorical variables, respectively.

*P* values were calculated using analysis of variance or Kruskal-Wallis test for continuous variables and chi-square test for categorical variables. All *P* values from global comparisons were <0.001, except for smoking status (*P* = 0.073).Abbreviations: BMI, body mass index; HbA1c, glycated hemoglobin A1c; SBP, systolic blood pressure; TC, total cholesterol; HDL-c, high-density lipoprotein cholesterol; eGFR, estimated glomerular filtration rate; CRP, C-reactive protein; SD, standard deviation; IQR, interquartile range.

During a median follow-up of 10.7 years (100,898 person-years), there were 1429 cases of incident dementia (14.16 per 1000 person-years). The incidence rates of dementia were 12.32 per 1000 person-years for normoglycemia, 14.98 per 1000 person-years for prediabetes, 14.00 per 1000 person-years for diabetes subgroup 1, 27.73 per 1000 person-years for diabetes subgroup 2, and 18.98 per 1000 person-years for diabetes subgroup 3. Kaplan-Meier curves for the cumulative incidence of dementia showed significant differences between different glycemic status and diabetes subgroups (log-rank *P* value <0.001) (**Supplementary Figure 4**). After covariates adjustment, the HRs (95% CIs) for incident dementia were 0.96 (0.85-1.09) for prediabetes, 1.10 (0.89-1.37) for diabetes subgroup 1, 1.06 (0.87-1.28) for diabetes subgroup 2, and 1.45 (1.17-1.80) for diabetes subgroup 3, compared to those with normoglycemia. After further adjusting for lifestyle factors, the HRs (95% CIs) for incident dementia were 0.96 (0.84-1.08) for prediabetes, 1.06 (0.85-1.31) for diabetes subgroup 1, 1.02 (0.84-1.24) for diabetes subgroup 2, and 1.43 (1.15-1.77) for diabetes subgroup 3 (**Table 2**).

**Table 2:** Risk of incident dementia according to different glycemic status and diabetes subgroups.

				Crude model		Model 1^*^		Model 2^†^	
Glycemic status and diabetes subgroups	No. of participants	No. of events/ person-years	Incidence rate per 1000 person-years	HR (95% CI)	*P* value	HR (95% CI)	*P* value	HR (95% CI)	*P* value
Normoglycemia	5999	723/58,682	12.32	1 [Reference]	–	1 [Reference]	–	1 [Reference]	–
Prediabetes	2711	379/25,295	14.98	1.23 (1.08, 1.39)	0.001	0.96 (0.85, 1.09)	0.547	0.96 (0.84, 1.08)	0.487
Diabetes (Subgroup 1)^‡^	784	98/7002	14.00	1.16 (0.94, 1.43)	0.170	1.10 (0.89, 1.37)	0.385	1.06 (0.85, 1.31)	0.627
Diabetes (Subgroup 2)^§^	631	129/4652	27.73	2.39 (1.98, 2.89)	<0.001	1.06 (0.87, 1.28)	0.579	1.02 (0.84, 1.24)	0.826
Diabetes (Subgroup 3)^||^	580	100/5268	18.98	1.57 (1.28, 1.94)	<0.001	1.45 (1.17, 1.80)	<0.001	1.43 (1.15, 1.77)	0.001

^*^ Model 1 was adjusted for age, sex, race, education level, marital status, hypertension, heart disease, stroke, lung disease, and cancer.

^†^ Model 2 was additionally adjusted for never smoking status, low to moderate alcohol consumption, and top tertile of physical activity.

^‡^ Subgroup 1: high BMI and elevated CRP.

^§^ Subgroup 2: late-onset diabetes, high blood pressure, and low eGFR.

^||^ Subgroup 3: early-onset diabetes, high HbA1c, and high cholesterol levels.Abbreviations: HR, hazard ratio; CI, confidence interval; BMI, body mass index; eGFR, estimated glomerular filtration rate; HbA1c, glycated hemoglobin A1c.

### 3.3 Association of lifestyle with incident dementia stratified by glycemic status and diabetes subgroups

The percentages of participants with a favorable lifestyle (lifestyle score 2–3 points) were 37.1% for normoglycemia, 30.8% for prediabetes, 18.9% for diabetes subgroup 1, 22.7% for diabetes subgroup 2, and 28.6% for diabetes subgroup 3. The association between lifestyle status and incident dementia in different glycemic status and diabetes subgroups is shown in **Table 3**. Compared to individuals with an unfavorable lifestyle (lifestyle score 0 points), the related HRs (95% CIs) regarding incident dementia among those with a favorable lifestyle were 0.70 (0.57-0.87) for normoglycemia, 0.67 (0.50-0.90) for prediabetes, 0.33 (0.14-0.79) for diabetes subgroup 1, 0.90 (0.55-1.49) for diabetes subgroup 2, and 0.77 (0.43-1.37) for diabetes subgroup 3. Each 1-category increment in lifestyle status was associated with a 16% reduction in dementia risk (HR 0.84, 95% CI 0.76-0.93) for normoglycemia, a 18% reduction (HR 0.82, 95% CI 0.71-0.95) for prediabetes, and a 29% reduction (HR 0.71, 95% CI 0.52-0.98) for diabetes subgroup 1. However, no statistically significant reduction was observed for diabetes subgroup 2 (HR 0.94, 95% CI 0.73-1.22) or subgroup 3 (HR 0.86, 95% CI 0.64-1.16) (**Table 3**). A similar pattern was observed with each 1-point lifestyle score increment, showing a 15% reduction in dementia risk (HR 0.85, 95% CI 0.78-0.94) for normoglycemia, 17% (HR 0.83, 95% CI 0.73-0.95) for prediabetes, and 27% (HR 0.73, 95% CI 0.55-0.99) for diabetes subgroup 1, but no statistically significant reductions were observed for subgroup 2 (HR 0.97, 95% CI 0.76-1.23) and subgroup 3 (HR 0.86, 95% CI 0.66-1.13) (**Table 3**).

**Table 3:** Association of healthy lifestyle with incident dementia in participants with different glycemic status and diabetes subgroups.

Lifestyle category	No. of participants	No. of events/ person-years	Incidence rate per 1000 person-years	HR (95% CI)^*^	*P* value
Normoglycemia					
Unfavorable lifestyle	1302	180/11,431	15.75	1 [Reference]	–
Intermediate lifestyle	2471	339/23,661	14.33	0.89 (0.74, 1.07)	0.231
Favorable lifestyle	2226	204/23,590	8.65	0.70 (0.57, 0.87)	<0.001
Per 1-category increment				0.84 (0.76, 0.93)	<0.001
Per 1-point score increment				0.85 (0.78, 0.94)	<0.001
Prediabetes					
Unfavorable lifestyle	604	98/5145	19.05	1 [Reference]	–
Intermediate lifestyle	1273	195/11,713	16.65	0.86 (0.68, 1.11)	0.246
Favorable lifestyle	834	86/8437	10.19	0.67 (0.50, 0.90)	0.008
Per 1-category increment				0.82 (0.71, 0.95)	0.008
Per 1-point score increment				0.83 (0.73, 0.95)	0.006
Diabetes (Subgroup 1)^†^					
Unfavorable lifestyle	274	40/2134	18.75	1 [Reference]	–
Intermediate lifestyle	362	52/3326	15.64	0.98 (0.63, 1.52)	0.940
Favorable lifestyle	148	6/1543	3.89	0.33 (0.14, 0.79)	0.013
Per 1-category increment				0.71 (0.52, 0.98)	0.038
Per 1-point score increment				0.73 (0.55, 0.99)	0.043
Diabetes (Subgroup 2)^‡^					
Unfavorable lifestyle	186	39/1253	31.13	1 [Reference]	–
Intermediate lifestyle	302	59/2161	27.30	0.82 (0.54, 1.27)	0.377
Favorable lifestyle	143	31/1238	25.04	0.90 (0.55, 1.49)	0.687
Per 1-category increment				0.94 (0.73, 1.22)	0.665
Per 1-point score increment				0.97 (0.76, 1.23)	0.798
Diabetes (Subgroup 3)^§^					
Unfavorable lifestyle	149	32/1233	25.96	1 [Reference]	–
Intermediate lifestyle	265	46/2392	19.23	0.74 (0.46, 1.20)	0.222
Favorable lifestyle	166	22/1643	13.39	0.77 (0.43, 1.37)	0.372
Per 1-category increment				0.86 (0.64, 1.16)	0.329
Per 1-point score increment				0.86 (0.66, 1.13)	0.282

^*^ Model was adjusted for age, sex, race, education level, marital status, hypertension, heart disease, stroke, lung disease, and cancer.

^†^ Subgroup 1: high BMI and elevated CRP.

^‡^ Subgroup 2: late-onset diabetes, high blood pressure, and low eGFR.

^§^ Subgroup 3: early-onset diabetes, high HbA1c, and high cholesterol levels.Abbreviations: HR, hazard ratio; CI, confidence interval; BMI, body mass index; eGFR, estimated glomerular filtration rate; HbA1c, glycated hemoglobin A1c.

### 3.4 Sensitivity analysis

The associations of diabetes subgroups and lifestyle status with incident dementia did not appreciably change after accounting for death as a competing risk, excluding dementia events occurring within the first three years of follow-up, applying IPCW to address potential informative censoring, additionally adjusting for depression, APOE-4 carrier status, and baseline TICS, or using Cox regression with Firth’s penalized likelihood to address sparse events (**Supplementary Tables 2**–**11**).

## 4 Discussion

### 4.1 Principal findings

Using data from a large, nationally representative cohort, we employed the SOM method and identified three diabetes subgroups among diabetes: high BMI and elevated CRP (subgroup 1); late-onset diabetes, high blood pressure, and low eGFR (subgroup 2); early-onset diabetes, high HbA1c, and high cholesterol levels (subgroup 3). Only subgroup 3 showed a significantly higher risk of dementia compared with normoglycemia. However, a favorable lifestyle was associated with a lower risk of dementia in subgroup 1, whereas no statistically significant associations were observed in subgroups 2 and 3.

### 4.2 Comparison with previous evidence

Effects of a combination of healthy lifestyle factors on dementia in patients with diabetes are increasingly discussed. A large prospective study with 167,946 participants from the UK Biobank cohort found that adhering to a broad range of healthy lifestyle behaviors was associated with a significantly lower risk of diabetes-related dementia (6). However, little is known about whether these effects vary across different subgroups of diabetes. In 2021, Bancks et al. characterized exclusive subgroups of diabetes based on fasting glucose, age at diabetes diagnosis, and waist circumference, and assessed the associated risk for dementia in the Multi-Ethnic Study of Atherosclerosis (MESA) (28). They found that the subgroup characterized by early-onset diabetes and high waist circumference had the highest risk of dementia. Later, they used a different approach to characterize diabetes subgroups and assessed the associations of lifestyle intervention with mild cognitive impairment (MCI)/probable dementia (PD) in different subgroups (29). They identified four diabetes subgroups characterized by older age at onset, high HbA1c, severe obesity, and younger age at onset, respectively (29). The severe obesity subgroup had the highest risk of dementia and the younger onset subgroup had the lowest. No evidence was found that lifestyle intervention was associated with a reduced risk of MCI/PD within any diabetes subgroup.

### 4.3 Implications for subgroup-specific dementia prevention

A major novel finding of our study is the identification of three diabetes subgroups with distinct risk-benefit profiles, which may have important implications for tailored prevention strategies. Subgroup 1 (high BMI and elevated CRP) did not exhibit an increased risk of dementia compared with normoglycemia. However, adherence to a healthy lifestyle can reduce the risk of dementia, suggesting that lifestyle may confer meaningful benefits in this subgroup. These findings highlight the need to prioritize and actively promote a healthy lifestyle in such individuals. In contrast, subgroup 3 (early-onset diabetes, high HbA1c, and high cholesterol levels) exhibited a significantly increased risk of dementia, whereas the association between a favorable lifestyle and incident dementia in this subgroup was not statistically significant and requires further investigation. Nevertheless, lifestyle modification may remain relevant as part of integrated management alongside pharmacological treatment and control of hyperglycemia, dyslipidemia, and other vascular risk factors in this subgroup. Future studies should examine whether the association between lifestyle and dementia risk varies according to treatment patterns and metabolic control.

### 4.4 Potential mechanisms underlying subgroup-specific lifestyle benefits

Previous studies have proposed several hypotheses regarding the mechanisms underlying the relationship between healthy lifestyle factors and reduced dementia risks, primarily involving reduced oxidative stress, anti-inflammatory effects, improved vascular health, and upregulation of neurotrophic factors (30). Given that patients in diabetes subgroup 1 exhibited elevated BMI and CRP levels, the observed favorable impact of a combination of healthy lifestyle factors on dementia in this subgroup may be explained by their role in lowering BMI and alleviating chronic systemic inflammation.

### 4.5 Prediabetes and dementia risk

Meanwhile, our study suggested that prediabetes was not associated with a higher dementia risk. Findings from previous studies on the relationship between prediabetes and dementia risk remain controversial. A meta-analysis of 144 prospective studies found that prediabetes was associated with a higher risk of dementia (31), while data from the Whitehall II study and the UK Biobank did not show evidence of such an association (6, 18). Differences in methodologies for assessing glucose status (e.g., HbA1c, fasting, or random blood glucose) and dementia outcomes (e.g., using ICD-10 codes or cognitive scores), and follow-up duration may partly explain these discrepancies.

### 4.6 Strengths and limitations

The strengths of our study include a large sample size and a nationally representative cohort with long-term follow-up. This allowed us to perform detailed analysis to explore dementia risk associated with different diabetes subgroups and also healthy lifestyle factors. More importantly, our study examined the prevention of dementia through healthy lifestyle management by categorizing patients with diabetes into distinct subgroups using advanced unsupervised statistical methods. However, our study also has some limitations. First, because the HRS did not measure fasting blood glucose, glycemic status was determined based on HbA1c levels and self-reported diagnosis, and this exposure misclassification potentially dilutes the observed associations. Second, lifestyle factors were assessed only at baseline, meaning that any changes in these factors over time were not taken into account. The inability to account for these changes could result in an underestimation or overestimation of their impact on long-term outcomes. In addition, information on other lifestyle factors, including diet, sleep, and sedentary behavior, was unavailable in the present analysis. This omission could introduce information bias regarding the lifestyle score and limit its interpretation as a comprehensive measure of healthy lifestyle. Future studies integrating a broader range of lifestyle factors are warranted. Third, although we adjusted for multiple confounding factors, including key personal characteristics and comorbidities, residual confounding from unmeasured confounders remains possible. In addition, reverse causality cannot be excluded due to the nature of observational studies. Fourth, although the three-cluster solution was consistently supported by different methods, the reproducibility and generalizability of the identified subgroups require confirmation in an independent population. Fifth, although identifying dementia on the basis of either a self-reported physician diagnosis of dementia or a TICS score ≤6 is pragmatic, the TICS remains a screening instrument rather than a diagnostic gold standard. Therefore, the potential for misclassification cannot be excluded, particularly among older or less educated participants. Finally, participants in the HRS were primarily of White ethnicity and community-dwelling adults, which limits the generalizability of our results to broader populations. Subgroup prevalence, phenotypic profiles, and dementia associations may differ in other settings. Subgroup 2-like phenotypes may be more common in clinical cohorts with a greater burden of complications, whereas subgroup 3-like phenotypes may be more common in cohorts with younger-onset diabetes. External validation in diverse independent cohorts is therefore warranted.

## 5 Conclusions

Using the SOM clustering analysis, we identified three diabetes subgroups with different clinical profiles, varying associations with dementia risk, and differing effects of a combination of healthy lifestyle factors. Our findings provide valuable insights into reducing the burden of dementia in specific diabetes subgroups and may help develop targeted lifestyle modifications to prevent dementia in patients with distinct characteristics.

## Data Availability

Publicly available datasets were analyzed in this study. This data can be found here: The original HRS dataset is available at https://hrs.isr.umich.edu/.
