# Supplementary material for: Associations of diabetes subgroups and lifestyle with incident dementia: insights from unsupervised phenotype clustering analysis
Source: Front Endocrinol (Lausanne). 2026 Sep 8;17:1938222. doi: 10.3389/fendo.2026.1938222 (PMC13593420; doi:10.3389/fendo.2026.1938222)
Supplement: Supplementary file 1 [file DataSheet1.pdf]

Supplementary material

Associations of diabetes subgroups and lifestyle with incident dementia: insights from unsupervised phenotype clustering analysis

Contents:

Table S1. Characteristics for the clustering variables in SOM analysis 2
Table S2. Risk of incident dementia according to different glycemic status and diabetes subgroups using a competing risk analysis 3
Table S3. Association of healthy lifestyle with incident dementia in participants with different glycemic status and diabetes subgroups using a competing risk analysis.......4
Table S4. Risk of incident dementia according to different glycemic status and diabetes subgroups after excluding events that occurred within the first three years of follow-up 6
Table S5. Association of healthy lifestyle with incident dementia in participants with different glycemic status and diabetes subgroups after excluding events that occurred within the first three years of follow-up 7
Table S6. Risk of incident dementia according to different glycemic status and diabetes subgroups using the IPCW Cox regression models 9
Table S7. Association of healthy lifestyle with incident dementia in participants with different glycemic status and diabetes subgroups using the IPCW Cox regression models 10
Table S8. Risk of incident dementia according to different glycemic status and diabetes subgroups after adjusting for depression, APOE-4 carrier status and baseline TICS..12
Table S9. Association of healthy lifestyle with incident dementia in participants with different glycemic status and diabetes subgroups after adjusting for depression, APOE-4 carrier status and baseline TICS...13
Table S10. Risk of incident dementia according to different glycemic status and diabetes subgroups using the Firth penalized method 15
Table S11. Association of healthy lifestyle with incident dementia in participants with different glycemic status and diabetes subgroups using the Firth penalized method ..16
Figure S1. Flowchart of study population..18
Figure S2. Results of model-based clustering...19
Figure S4. Kaplan-Meier plots for dementia in different glycemic status and diabetes subgroups 21

Table S1. Characteristics for the clustering variables in SOM analysis

Characteristics	Subgroup 1* (n = 898)	Subgroup 2† (n = 731)	Subgroup 3‡ (n = 663)	*P* value
Age at onset of diabetes, years	57.3 (10.2)	69.3 (9.9)	54.6 (10.8)	<0.001
BMI, kg/m^2	36.2 (6.7)	28.8 (4.7)	30.0 (5.3)	<0.001
HbA1c, %	6.7 (1.0)	6.6 (0.8)	7.8 (2.0)	<0.001
SBP, mmHg	129.0 (21.0)	139.6 (21.0)	134.0 (19.6)	<0.001
TC, mg/dL	185.2 (35.2)	182.9 (40.0)	213.8 (42.3)	<0.001
HDL-c, mg/dL	45.2 (10.6)	46.8 (12.6)	60.5 (16.3)	<0.001
eGFR, mL/min/1.73 m^2	65.5 (25.8)	51.4 (20.3)	78.4 (23.4)	<0.001
CRP, mg/L	5.5 (2.4-10.8)	1.6 (0.8-3.8)	1.7 (0.8-3.7)	<0.001

* Subgroup 1: high BMI and elevated CRP.

† Subgroup 2: late-onset diabetes, high blood pressure, and low eGFR.

‡ Subgroup 3: early-onset diabetes, high HbA1c, and high cholesterol levels.

Data are mean (SD) or median (IQR).

P values were calculated using analysis of variance or Kruskal-Wallis test.

Abbreviations: SOM, self-organizing maps; BMI, body mass index; HbA1c, hemoglobin A1c; SBP, systolic blood pressure; TC, total cholesterol; HDL-c, high-density lipoprotein cholesterol; eGFR, estimated glomerular filtration rate; CRP, C-reactive protein; SD, standard deviation; IQR, interquartile range.

Table S2. Risk of incident dementia according to different glycemic status and diabetes subgroups using a competing risk analysis

Glycemic status and diabetes subgroups	No. of participants	No. of events/ person-years	Incidence rate per 1000 person-years	SHR (95% CI)*	*P* value
Normoglycemia	5999	723/58,682	12.32	1 [Reference]	-
Prediabetes	2711	379/25,295	14.98	0.96 (0.85, 1.09)	0.540
Diabetes (Subgroup 1)†	784	98/7002	14.00	0.95 (0.77, 1.18)	0.670
Diabetes (Subgroup 2)‡	631	129/4652	27.73	0.97 (0.80, 1.19)	0.790
Diabetes (Subgroup 3)§	580	100/5268	18.98	1.32 (1.06, 1.64)	0.012

* Model was adjusted for age, sex, race, education level, marital status, never smoking status, low to moderate alcohol consumption, high physical activity, hypertension, heart disease, stroke, lung disease, and cancer.

† Subgroup 1: high BMI and elevated CRP.

‡ Subgroup 2: late-onset diabetes, high blood pressure, and low eGFR.

§ Subgroup 3: early-onset diabetes, high HbA1c, and high cholesterol levels.

Abbreviations: SHR, subdistribution hazard ratio; CI, confidence interval; BMI, body mass index; CRP, C-reactive protein; eGFR, estimated glomerular filtration rate; HbA1c, hemoglobin A1c.

Table S3. Association of healthy lifestyle with incident dementia in participants with different glycemic status and diabetes subgroups using a competing risk analysis

Lifestyle category	No. of participants	No. of events/ person-years	Incidence rate per 1000 person-years	SHR (95% CI)*	*P* value
Normoglycemia					
Unfavorable lifestyle	1302	180/11,431	15.75	1 [Reference]	-
Intermediate lifestyle	2471	339/23,661	14.33	0.98 (0.81, 1.19)	0.830
Favorable lifestyle	2226	204/23,590	8.65	0.84 (0.68, 1.04)	0.100
Per 1-category increment				0.91 (0.83, 1.01)	0.088
Per 1-point score increment				0.92 (0.84, 1.01)	0.086
Prediabetes					
Unfavorable lifestyle	604	98/5145	19.05	1 [Reference]	-
Intermediate lifestyle	1273	195/11,713	16.65	0.92 (0.71, 1.18)	0.490
Favorable lifestyle	834	86/8437	10.19	0.78 (0.57, 1.05)	0.100
Per 1-category increment				0.88 (0.76, 1.02)	0.100
Per 1-point score increment				0.89 (0.78, 1.02)	0.083
Diabetes (Subgroup 1)†					
Unfavorable lifestyle	274	40/2134	18.75	1 [Reference]	-
Intermediate lifestyle	362	52/3326	15.64	1.09 (0.71, 1.67)	0.690
Favorable lifestyle	148	6/1543	3.89	0.43 (0.17, 1.06)	0.066
Per 1-category increment				0.81 (0.61, 1.10)	0.180
Per 1-point score increment				0.83 (0.62, 1.11)	0.220
Diabetes (Subgroup 2)‡					
Unfavorable lifestyle	186	39/1253	31.13	1 [Reference]	-
Intermediate lifestyle	302	59/2161	27.30	0.94 (0.61, 1.43)	0.760

Favorable lifestyle	143	31/1238	25.04	1.16 (0.71, 1.89)	0.550
Per 1-category increment				1.07 (0.83, 1.38)	0.610
Per 1-point score increment				1.09 (0.86, 1.39)	0.470
Diabetes (Subgroup 3)§					
Unfavorable lifestyle	149	32/1233	25.96	1 [Reference]	-
Intermediate lifestyle	265	46/2392	19.23	0.80 (0.50, 1.28)	0.360
Favorable lifestyle	166	22/1643	13.39	0.83 (0.48, 1.45)	0.520
Per 1-category increment				0.90 (0.68, 1.20)	0.480
Per 1-point score increment				0.89 (0.69, 1.16)	0.390

* Model was adjusted for age, sex, race, education level, marital status, hypertension, heart disease, stroke, lung disease, and cancer.

† Subgroup 1: high BMI and elevated CRP.

‡ Subgroup 2: late-onset diabetes, high blood pressure, and low eGFR.

§ Subgroup 3: early-onset diabetes, high HbA1c, and high cholesterol levels.

Abbreviations: SHR, subdistribution hazard ratio; CI, confidence interval; BMI, body mass index; CRP, C-reactive protein; eGFR, estimated glomerular filtration rate; HbA1c, hemoglobin A1c.

Table S4. Risk of incident dementia according to different glycemic status and diabetes subgroups after excluding events that occurred within the first three years of follow-up

Glycemic status and diabetes subgroups	No. of participants	No. of events/ person-years	Incidence rate per 1000 person-years	HR (95% CI)*	*P* value
Normoglycemia	5861	585/58,366	10.02	1 [Reference]	-
Prediabetes	2630	298/25,110	11.87	0.95 (0.83, 1.09)	0.485
Diabetes (Subgroup 1)†	762	76/6951	10.93	1.07 (0.84, 1.37)	0.596
Diabetes (Subgroup 2)‡	611	109/4605	23.67	1.14 (0.93, 1.41)	0.210
Diabetes (Subgroup 3)§	559	79/5220	15.13	1.49 (1.17, 1.90)	0.001

* Model was adjusted for age, sex, race, education level, marital status, never smoking status, low to moderate alcohol consumption, high physical activity, hypertension, heart disease, stroke, lung disease, and cancer.

† Subgroup 1: high BMI and elevated CRP.

‡ Subgroup 2: late-onset diabetes, high blood pressure, and low eGFR.

§ Subgroup 3: early-onset diabetes, high HbA1c, and high cholesterol levels.

Abbreviations: HR, hazard ratio; CI, confidence interval; BMI, body mass index; CRP, C-reactive protein; eGFR, estimated glomerular filtration rate; HbA1c, hemoglobin A1c.

Table S5. Association of healthy lifestyle with incident dementia in participants with different glycemic status and diabetes subgroups after excluding events that occurred within the first three years of follow-up

Lifestyle category	No. of participants	No. of events/ person-years	Incidence rate per 1000 person-years	HR (95% CI)*	*P* value
Normoglycemia					
Unfavorable lifestyle	1254	132/11,322	11.66	1 [Reference]	-
Intermediate lifestyle	2402	270/23,506	11.49	0.95 (0.76, 1.17)	0.605
Favorable lifestyle	2205	183/23,538	7.77	0.79 (0.63, 1.00)	0.053
Per 1-category increment				0.89 (0.79, 1.00)	0.041
Per 1-score increment				0.90 (0.81, 0.99)	0.032
Prediabetes					
Unfavorable lifestyle	576	70/5081	13.78	1 [Reference]	-
Intermediate lifestyle	1236	158/11,628	13.59	0.96 (0.73, 1.28)	0.804
Favorable lifestyle	818	70/8401	8.33	0.70 (0.50, 0.99)	0.042
Per 1-category increment				0.84 (0.71, 0.99)	0.037
Per 1-score increment				0.85 (0.73, 0.98)	0.028
Diabetes (Subgroup 1)†					
Unfavorable lifestyle	265	31/2113	14.67	1 [Reference]	-
Intermediate lifestyle	351	41/3300	12.42	0.95 (0.58, 1.57)	0.841
Favorable lifestyle	146	4/1538	2.60	0.24 (0.08, 0.70)	0.009
Per 1-category increment				0.65 (0.45, 0.93)	0.020
Per 1-score increment				0.68 (0.49, 0.96)	0.027
Diabetes (Subgroup 2)‡					
Unfavorable lifestyle	182	35/1243	28.17	1 [Reference]	-
Intermediate lifestyle	290	47/2133	22.04	0.70 (0.44, 1.12)	0.141

Favorable lifestyle	139	27/1229	21.96	0.81 (0.47, 1.39)	0.445
Per 1-category increment				0.89 (0.67, 1.18)	0.424
Per 1-score increment				0.90 (0.69, 1.16)	0.413
Diabetes (Subgroup 3)[§]					
Unfavorable lifestyle	142	25/1219	20.51	1 [Reference]	-
Intermediate lifestyle	254	35/2366	14.79	0.74 (0.43, 1.27)	0.277
Favorable lifestyle	163	19/1635	11.62	0.77 (0.41, 1.44)	0.412
Per 1-category increment				0.87 (0.63, 1.20)	0.386
Per 1-score increment				0.87 (0.64, 1.17)	0.352

[*] Model was adjusted for age, sex, race, education level, marital status, hypertension, heart disease, stroke, lung disease, and cancer.

[†] Subgroup 1: high BMI and elevated CRP.

[‡] Subgroup 2: late-onset diabetes, high blood pressure, and low eGFR.

[§] Subgroup 3: early-onset diabetes, high HbA1c, and high cholesterol levels.

Abbreviations: HR, hazard ratio; CI, confidence interval; BMI, body mass index; CRP, C-reactive protein; eGFR, estimated glomerular filtration rate; HbA1c, hemoglobin A1c.

Table S6. Risk of incident dementia according to different glycemic status and diabetes subgroups using the IPCW Cox regression models

Glycemic status and diabetes subgroups	No. of participants	No. of events/ person-years	Incidence rate per 1000 person-years	HR (95% CI)*	*P* value
Normoglycemia	5999	723/58,682	12.32	1 [Reference]	-
Prediabetes	2711	379/25,295	14.98	0.96 (0.84, 1.09)	0.513
Diabetes (Subgroup 1)†	784	98/7002	14.00	1.04 (0.84, 1.29)	0.740
Diabetes (Subgroup 2)‡	631	129/4652	27.73	1.03 (0.84, 1.25)	0.804
Diabetes (Subgroup 3)§	580	100/5268	18.98	1.40 (1.13, 1.74)	0.002

* Model was adjusted for age, sex, race, education level, marital status, never smoking status, low to moderate alcohol consumption, high physical activity, hypertension, heart disease, stroke, lung disease, and cancer.
† Subgroup 1: high BMI and elevated CRP.
‡ Subgroup 2: late-onset diabetes, high blood pressure, and low eGFR.
§ Subgroup 3: early-onset diabetes, high HbA1c, and high cholesterol levels.
Abbreviations: IPCW, inverse probability of censoring weighted; HR, hazard ratio; CI, confidence interval; BMI, body mass index; CRP, C-reactive protein; eGFR, estimated glomerular filtration rate; HbA1c, hemoglobin A1c.

Table S7. Association of healthy lifestyle with incident dementia in participants with different glycemic status and diabetes subgroups using the IPCW Cox regression models

Lifestyle category	No. of participants	No. of events/ person-years	Incidence rate per 1000 person-years	HR (95% CI)[*]	*P* value
Normoglycemia					
Unfavorable lifestyle	1302	180/11,431	15.75	1 [Reference]	-
Intermediate lifestyle	2471	339/23,661	14.33	0.89 (0.74, 1.08)	0.235
Favorable lifestyle	2226	204/23,590	8.65	0.70 (0.56, 0.86)	<0.001
Per 1-category increment				0.83 (0.75, 0.92)	<0.001
Per 1-point score increment				0.85 (0.77, 0.93)	<0.001
Prediabetes					
Unfavorable lifestyle	604	98/5145	19.05	1 [Reference]	-
Intermediate lifestyle	1273	195/11,713	16.65	0.88 (0.68, 1.12)	0.297
Favorable lifestyle	834	86/8437	10.19	0.67 (0.50, 0.90)	0.008
Per 1-category increment				0.82 (0.71, 0.95)	0.008
Per 1-point score increment				0.83 (0.73, 0.95)	0.005
Diabetes (Subgroup 1)[†]					
Unfavorable lifestyle	274	40/2134	18.75	1 [Reference]	-
Intermediate lifestyle	362	52/3326	15.64	0.97 (0.63, 1.49)	0.887
Favorable lifestyle	148	6/1543	3.89	0.35 (0.14, 0.87)	0.024
Per 1-category increment				0.72 (0.53, 0.98)	0.039
Per 1-point score increment				0.75 (0.55, 1.01)	0.056
Diabetes (Subgroup 2)[‡]					
Unfavorable lifestyle	186	39/1253	31.13	1 [Reference]	-
Intermediate lifestyle	302	59/2161	27.30	0.85 (0.55, 1.33)	0.477

Favorable lifestyle	143	31/1238	25.04	0.91 (0.56, 1.49)	0.720
Per 1-category increment				0.94 (0.73, 1.22)	0.665
Per 1-point score increment				0.98 (0.77, 1.24)	0.835
Diabetes (Subgroup 3)§					
Unfavorable lifestyle	149	32/1233	25.96	1 [Reference]	-
Intermediate lifestyle	265	46/2392	19.23	0.77 (0.48, 1.22)	0.263
Favorable lifestyle	166	22/1643	13.39	0.77 (0.44, 1.35)	0.358
Per 1-category increment				0.86 (0.64, 1.16)	0.329
Per 1-point score increment				0.86 (0.66, 1.12)	0.269

* Model was adjusted for age, sex, race, education level, marital status, hypertension, heart disease, stroke, lung disease, and cancer.

† Subgroup 1: high BMI and elevated CRP.

‡ Subgroup 2: late-onset diabetes, high blood pressure, and low eGFR.

§ Subgroup 3: early-onset diabetes, high HbA1c, and high cholesterol levels.

Abbreviations: IPCW, inverse probability of censoring weighted; HR, hazard ratio; CI, confidence interval; BMI, body mass index; CRP, C-reactive protein; eGFR, estimated glomerular filtration rate; HbA1c, hemoglobin A1c.

Table S8. Risk of incident dementia according to different glycemic status and diabetes subgroups after adjusting for depression, APOE-4 carrier status and baseline TICS

Glycemic status and diabetes subgroups	No. of participants	No. of events/ person-years	Incidence rate per 1000 person-years	HR (95% CI)*	*P* value
Normoglycemia	5999	723/58,682	12.32	1 [Reference]	-
Prediabetes	2711	379/25,295	14.98	1.01 (0.89, 1.14)	0.905
Diabetes (Subgroup 1)†	784	98/7002	14.00	1.02 (0.82, 1.26)	0.891
Diabetes (Subgroup 2)‡	631	129/4652	27.73	0.98 (0.81, 1.19)	0.852
Diabetes (Subgroup 3)§	580	100/5268	18.98	1.36 (1.09, 1.68)	0.005

* Model was adjusted for age, sex, race, education level, marital status, never smoking status, low to moderate alcohol consumption, high physical activity, hypertension, heart disease, stroke, lung disease, cancer, depression, APOE-4 carrier status, and baseline TICS.
† Subgroup 1: high BMI and elevated CRP.
‡ Subgroup 2: late-onset diabetes, high blood pressure, and low eGFR.
§ Subgroup 3: early-onset diabetes, high HbA1c, and high cholesterol levels.
Abbreviations: APOE, apolipoprotein E; TICS, Telephone Interview for Cognitive Status; HR, hazard ratio; CI, confidence interval; BMI, body mass index; CRP, C-reactive protein; eGFR, estimated glomerular filtration rate; HbA1c, hemoglobin A1c.

Table S9. Association of healthy lifestyle with incident dementia in participants with different glycemic status and diabetes subgroups after adjusting for depression, APOE-4 carrier status and baseline TICS

Lifestyle category	No. of participants	No. of events/ person-years	Incidence rate per 1000 person-years	HR (95% CI)*	*P* value
Normoglycemia					
Unfavorable lifestyle	1302	180/11,431	15.75	1 [Reference]	-
Intermediate lifestyle	2471	339/23,661	14.33	0.91 (0.76, 1.10)	0.325
Favorable lifestyle	2226	204/23,590	8.65	0.82 (0.66, 1.01)	0.060
Per 1-category increment				0.90 (0.81, 1.00)	0.059
Per 1-point score increment				0.91 (0.83, 1.00)	0.057
Prediabetes					
Unfavorable lifestyle	604	98/5145	19.05	1 [Reference]	-
Intermediate lifestyle	1273	195/11,713	16.65	0.88 (0.69, 1.12)	0.297
Favorable lifestyle	834	86/8437	10.19	0.79 (0.59, 1.07)	0.133
Per 1-category increment				0.89 (0.77, 1.04)	0.131
Per 1-point score increment				0.89 (0.78, 1.01)	0.078
Diabetes (Subgroup 1)†					
Unfavorable lifestyle	274	40/2134	18.75	1 [Reference]	-
Intermediate lifestyle	362	52/3326	15.64	1.06 (0.69, 1.64)	0.794
Favorable lifestyle	148	6/1543	3.89	0.39 (0.16, 0.96)	0.040
Per 1-category increment				0.79 (0.57, 1.08)	0.140
Per 1-point score increment				0.80 (0.59, 1.07)	0.130
Diabetes (Subgroup 2)‡					
Unfavorable lifestyle	186	39/1253	31.13	1 [Reference]	-
Intermediate lifestyle	302	59/2161	27.30	0.83 (0.53, 1.30)	0.414

Favorable lifestyle	143	31/1238	25.04	0.88 (0.53, 1.48)	0.640
Per 1-category increment				0.94 (0.72, 1.22)	0.644
Per 1-point score increment				0.98 (0.77, 1.25)	0.868
Diabetes (Subgroup 3)[§]					
Unfavorable lifestyle	149	32/1233	25.96	1 [Reference]	-
Intermediate lifestyle	265	46/2392	19.23	0.81 (0.50, 1.31)	0.391
Favorable lifestyle	166	22/1643	13.39	0.91 (0.52, 1.61)	0.743
Per 1-category increment				0.94 (0.70, 1.26)	0.671
Per 1-point score increment				0.92 (0.70, 1.19)	0.518

[*] Model was adjusted for age, sex, race, education level, marital status, hypertension, heart disease, stroke, lung disease, cancer, depression, APOE-4 carrier status, and baseline TICS.

[†] Subgroup 1: high BMI and elevated CRP.

[‡] Subgroup 2: late-onset diabetes, high blood pressure, and low eGFR.

[§] Subgroup 3: early-onset diabetes, high HbA1c, and high cholesterol levels.

Abbreviations: APOE, apolipoprotein E; TICS, Telephone Interview for Cognitive Status; HR, hazard ratio; CI, confidence interval; BMI, body mass index; CRP, C-reactive protein; eGFR, estimated glomerular filtration rate; HbA1c, hemoglobin A1c.

Table S10. Risk of incident dementia according to different glycemic status and diabetes subgroups using the Firth penalized method

Glycemic status and diabetes subgroups	No. of participants	No. of events/ person-years	Incidence rate per 1000 person-years	HR (95% CI)*	*P* value
Normoglycemia	5999	723/58,682	12.32	1 [Reference]	-
Prediabetes	2711	379/25,295	14.98	0.96 (0.84, 1.08)	0.494
Diabetes (Subgroup 1)†	784	98/7002	14.00	1.06 (0.85, 1.31)	0.604
Diabetes (Subgroup 2)‡	631	129/4652	27.73	1.02 (0.84, 1.24)	0.803
Diabetes (Subgroup 3)§	580	100/5268	18.98	1.44 (1.15, 1.77)	0.002

* Model was adjusted for age, sex, race, education level, marital status, never smoking status, low to moderate alcohol consumption, high physical activity, hypertension, heart disease, stroke, lung disease, and cancer.
† Subgroup 1: high BMI and elevated CRP.
‡ Subgroup 2: late-onset diabetes, high blood pressure, and low eGFR.
§ Subgroup 3: early-onset diabetes, high HbA1c, and high cholesterol levels.
Abbreviations: HR, hazard ratio; CI, confidence interval; BMI, body mass index; CRP, C-reactive protein; eGFR, estimated glomerular filtration rate; HbA1c, hemoglobin A1c.

Table S11. Association of healthy lifestyle with incident dementia in participants with different glycemic status and diabetes subgroups using the Firth penalized method

Lifestyle category	No. of participants	No. of events/ person-years	Incidence rate per 1000 person-years	HR (95% CI)*	*P* value
Normoglycemia					
Unfavorable lifestyle	1302	180/11,431	15.75	1 [Reference]	-
Intermediate lifestyle	2471	339/23,661	14.33	0.89 (0.74, 1.07)	0.227
Favorable lifestyle	2226	204/23,590	8.65	0.70 (0.57, 0.87)	<0.001
Per 1-category increment				0.84 (0.76, 0.93)	<0.001
Per 1-point score increment				0.85 (0.78, 0.94)	<0.001
Prediabetes					
Unfavorable lifestyle	604	98/5145	19.05	1 [Reference]	-
Intermediate lifestyle	1273	195/11,713	16.65	0.86 (0.68, 1.11)	0.242
Favorable lifestyle	834	86/8437	10.19	0.67 (0.50, 0.90)	0.008
Per 1-category increment				0.82 (0.71, 0.95)	0.008
Per 1-point score increment				0.83 (0.73, 0.95)	0.006
Diabetes (Subgroup 1)†					
Unfavorable lifestyle	274	40/2134	18.75	1 [Reference]	-
Intermediate lifestyle	362	52/3326	15.64	0.98 (0.64, 1.52)	0.929
Favorable lifestyle	148	6/1543	3.89	0.35 (0.14, 0.77)	0.007
Per 1-category increment				0.72 (0.52, 0.98)	0.038
Per 1-point score increment				0.74 (0.55, 0.99)	0.042
Diabetes (Subgroup 2)‡					
Unfavorable lifestyle	186	39/1253	31.13	1 [Reference]	-
Intermediate lifestyle	302	59/2161	27.30	0.82 (0.54, 1.27)	0.368

Favorable lifestyle	143	31/1238	25.04	0.90 (0.55, 1.49)	0.693
Per 1-category increment				0.95 (0.73, 1.22)	0.667
Per 1-point score increment				0.97 (0.76, 1.23)	0.811
Diabetes (Subgroup 3)§					
Unfavorable lifestyle	149	32/1233	25.96	1 [Reference]	-
Intermediate lifestyle	265	46/2392	19.23	0.74 (0.47, 1.20)	0.218
Favorable lifestyle	166	22/1643	13.39	0.78 (0.44, 1.36)	0.379
Per 1-category increment				0.87 (0.65, 1.16)	0.332
Per 1-point score increment				0.86 (0.66, 1.13)	0.289

* Model was adjusted for age, sex, race, education level, marital status, hypertension, heart disease, stroke, lung disease, and cancer.

† Subgroup 1: high BMI and elevated CRP.

‡ Subgroup 2: late-onset diabetes, high blood pressure, and low eGFR.

§ Subgroup 3: early-onset diabetes, high HbA1c, and high cholesterol levels.

Abbreviations: HR, hazard ratio; CI, confidence interval; BMI, body mass index; CRP, C-reactive protein; eGFR, estimated glomerular filtration rate; HbA1c, hemoglobin A1c.

Figure S1. Flowchart of study population

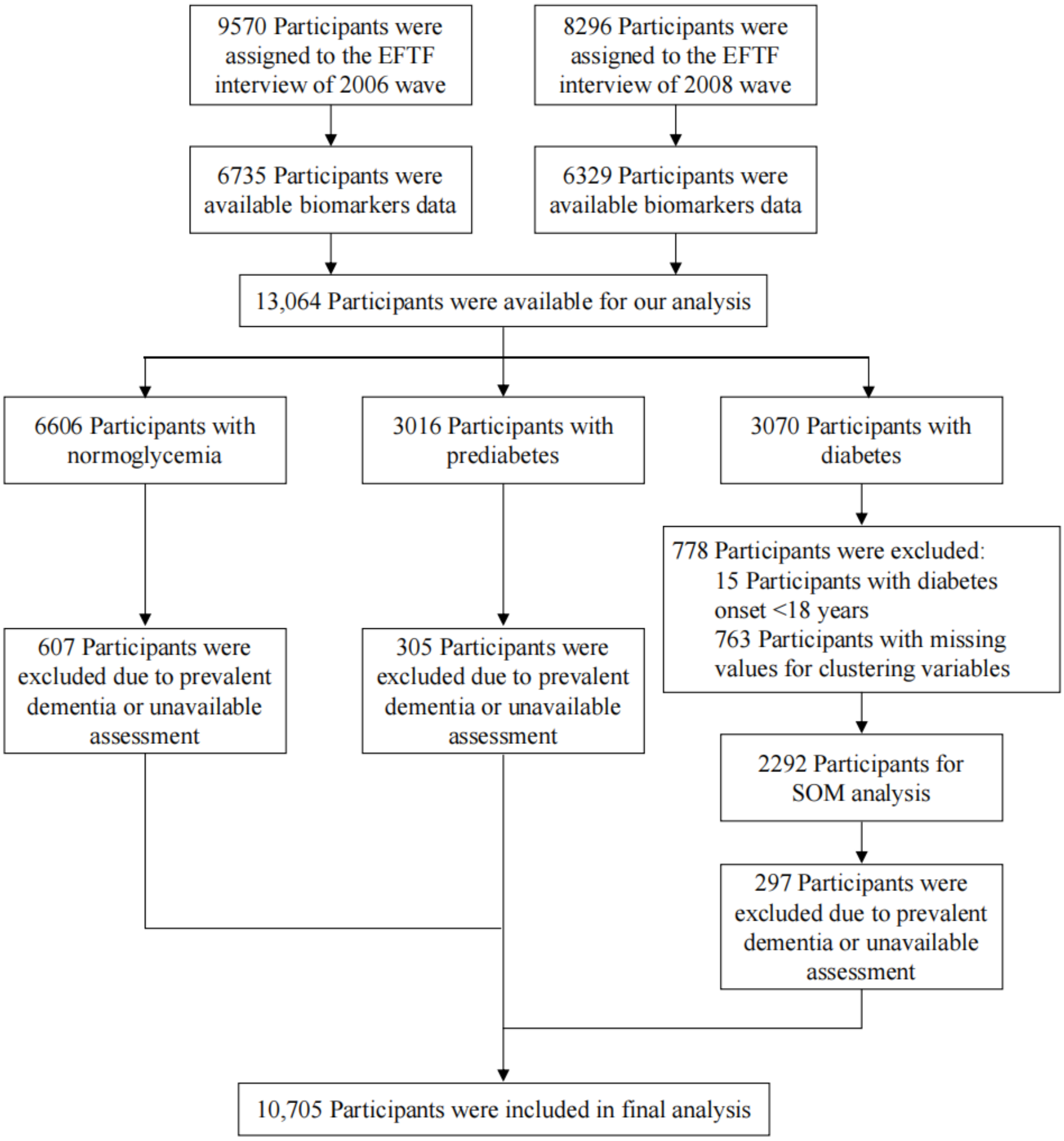

Figure S2. Results of model-based clustering

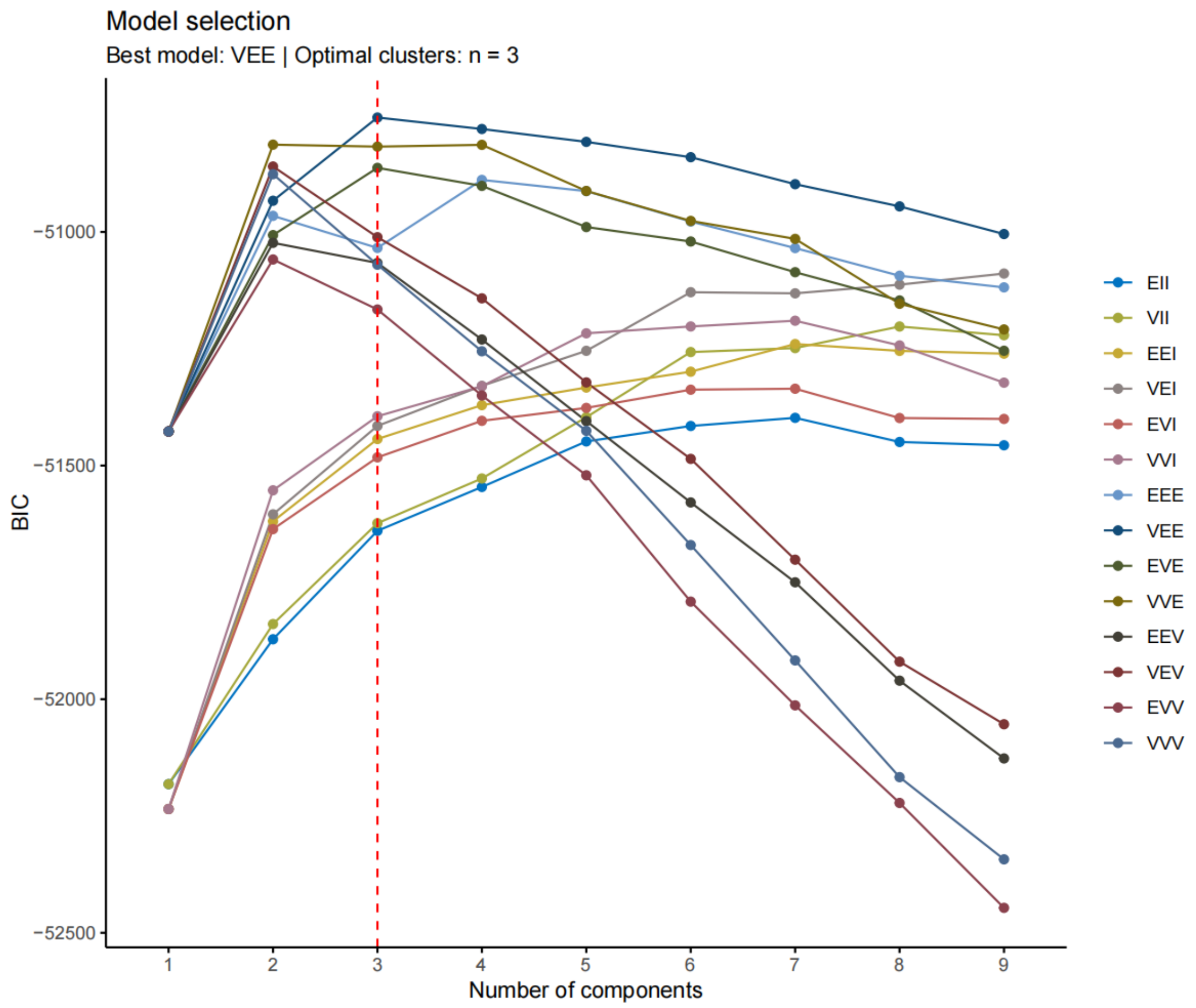

The combination of the three letters EVI represents 14 possible clustering models. Each of the volume, shape and orientation of the clusters can be constrained to be equal (E) or allowed to vary (V); the orientation can also be spherical, with identical eigenvalues (I). The BIC favors 3 groups.
BIC, Bayesian Information Criterion.

Figure S3. Results of NbClust consensus method

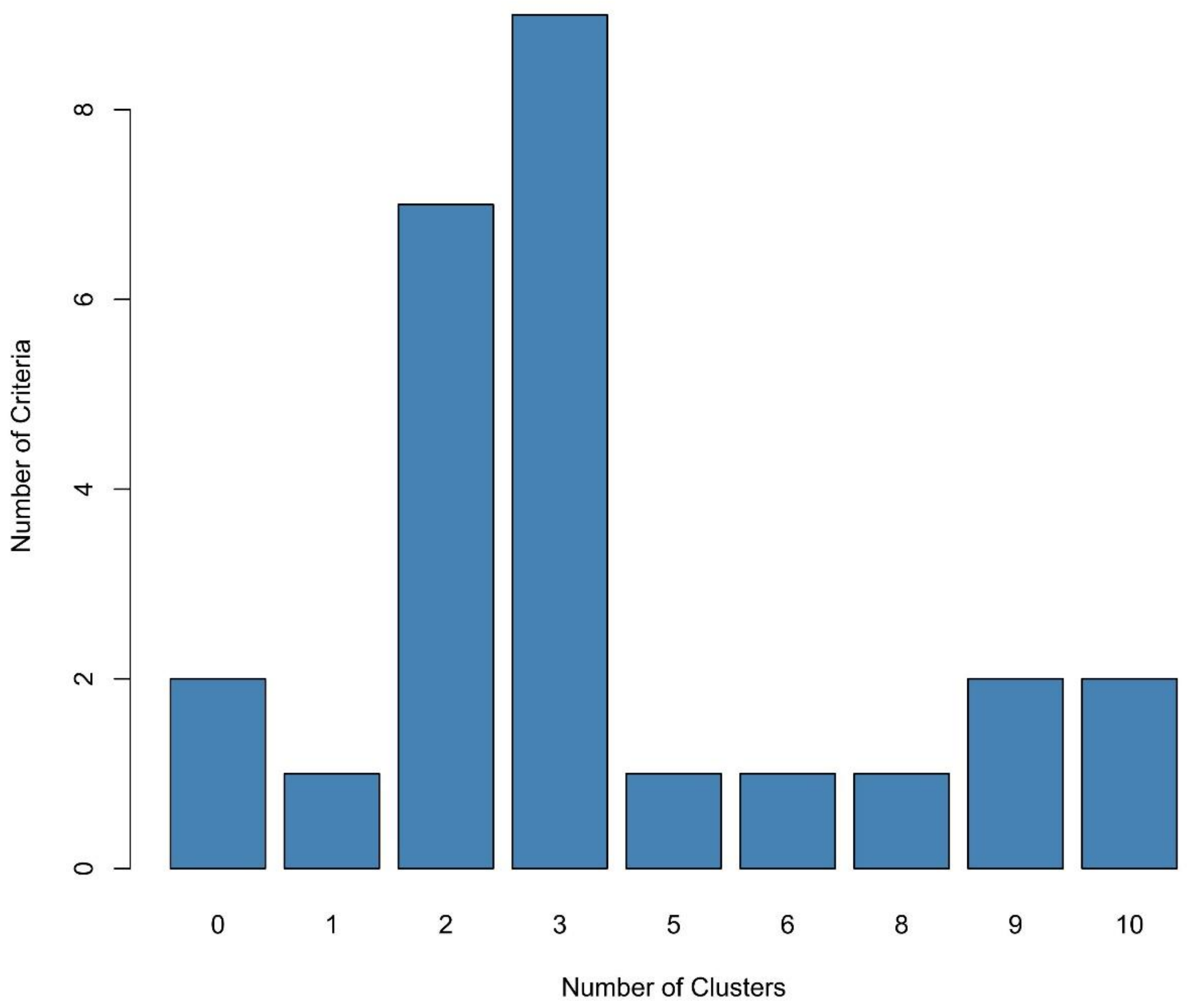

Candidate solutions were evaluated using k-means clustering with Euclidean distance. Bars indicate the number of internal validity indices recommending each solution. The three-cluster solution received the largest number of recommendations (9 indices).

Figure S4. Kaplan-Meier plots for dementia in different glycemic status and diabetes subgroups

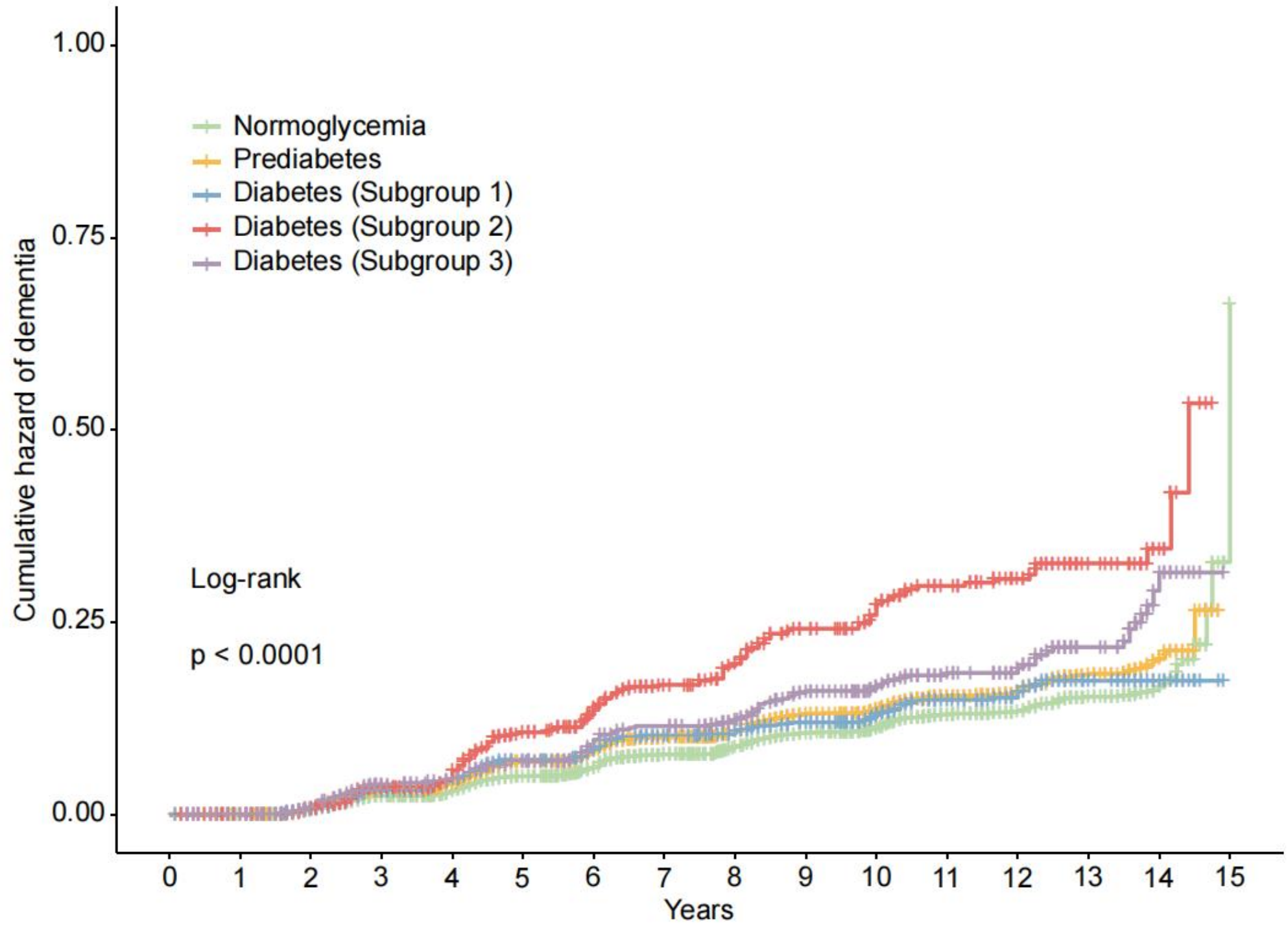

Number at risk

	0	1	2	3	4	5	6	7	8	9	10	11	12	13	14	15
Normoglycemia	5999	5929	5723	5421	5243	4905	4666	4387	4134	3767	3524	3161	2872	1567	630	2
Prediabetes	2711	2666	2588	2423	2321	2132	2007	1875	1750	1561	1454	1299	1136	537	245	0
Diabetes (Subgroup 1)	784	766	724	677	642	593	550	513	486	442	396	344	307	144	57	0
Diabetes (Subgroup 2)	631	611	572	509	478	410	365	322	284	229	200	166	137	49	19	0
Diabetes (Subgroup 3)	580	568	537	497	478	450	415	400	376	326	294	259	233	114	30	0

Subgroup 1: high BMI and elevated CRP.
Subgroup 2: late-onset diabetes, high blood pressure, and low eGFR.
Subgroup 3: early-onset diabetes, high HbA1c, and high cholesterol levels.
BMI, body mass index; CRP, C-reactive protein; eGFR, estimated glomerular filtration rate; HbA1c, hemoglobin A1c.
